# Regulation of IP_3_ receptors by cyclic AMP

**DOI:** 10.1016/j.ceca.2016.10.005

**Published:** 2017-05

**Authors:** Colin W. Taylor

**Affiliations:** Department of Pharmacology, University of Cambridge, Tennis Court Road, Cambridge CB2 1PD, UK

**Keywords:** AC, adenylyl cyclase, EPAC, exchange protein activated by cAMP, IP_3_, inositol 1,4,5-trisphosphate, IP_3_R, IP_3_ receptor, IRAG, IP_3_R-associated cGMP kinase substrate, IRBIT, IP_3_R-binding protein released by IP_3_, PKA, protein kinase A (cAMP-dependent protein kinase), PKG, protein kinase G (cGMP-dependent protein kinase), PLC, phospholipase C, *P*_o_, single-channel open probability, PTH, parathyroid hormone, Ca^2+^ stores, Cyclic AMP, IP_3_ receptor, Protein kinase A, Parathyroid hormone, Signalling junctions

## Abstract

Ca^2+^ and cAMP are ubiquitous intracellular messengers and interactions between them are commonplace. Here the effects of cAMP on inositol 1,4,5-trisphosphate receptors (IP_3_Rs) are briefly reviewed. All three subtypes of IP_3_R are phosphorylated by cAMP-dependent protein kinase (PKA). This potentiates IP_3_-evoked Ca^2+^ release through IP_3_R1 and IP_3_R2, but probably has little effect on IP_3_R3. In addition, cAMP can directly sensitize all three IP_3_R subtypes to IP_3_. The high concentrations of cAMP required for this PKA-independent modulation of IP_3_Rs is delivered to them within signalling junctions that include type 6 adenylyl cyclase and IP_3_R2.

## Highlights

• Inositol 1,4,5-trisphosphate receptors (IP_3_Rs) are modulated by cAMP.
• Phosphorylation by cAMP-dependent protein kinase (PKA) potentiates IP_3_-evoked Ca^2+^ release through IP_3_R1 and IP_3_R2.
• Delivery of cAMP to IP_3_R2s within signalling junctions directly potentiates their responses to IP_3_.

## Graphical abstract

**Figure:**
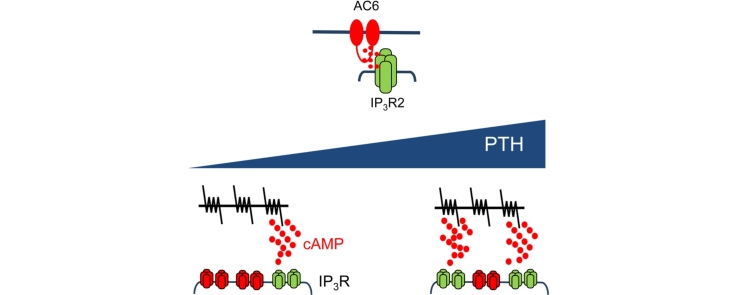

## 1 Introduction

Cyclic AMP and Ca^2+^ are ubiquitous intracellular messengers used by all eukaryotic cells from plants and animals to coordinate their behaviours in response to both extracellular signals and intracellular activity [1], [2], [3]. These messengers create a signalling ‘bottleneck’ through which many extracellular signals funnel to regulate diverse cellular responses. The capacity of a rather limited repertoire of intracellular messengers to selectively regulate cellular activities depends in large part on the spatial organization of the messengers within the cell, the time frames over which they are delivered, and interactions between messengers. The latter often endows signalling pathways with capacities to function as coincidence detectors: conveying signals onward only when several conditions are met [4]. As might be expected of the prototypical intracellular messengers, analyses of the interactions between cAMP and Ca^2+^ have a long history [5], [6] that has revealed interactions at many levels. Ca^2+^, for example, regulates formation and degradation of cAMP [2], [7], and cAMP can regulate both the channels that allow Ca^2+^ to flow into the cytosol and the Ca^2+^ pumps that extrude it [8], [9].

A ubiquitous pathway from extracellular stimuli to cytosolic Ca^2+^ signals is provided by receptors that stimulate phospholipase C (PLC), production of inositol 1,4,5-trisphosphate (IP_3_) and thereby Ca^2+^ release through IP_3_ receptors (IP_3_R) [10]. Cyclic AMP also modulates this pathway by, for example, regulating PLC [11] and the coupling of receptors to PLC [12]. However, in this short review, I focus on just one level of interaction, that between cAMP and IP_3_Rs [13], [14]. IP_3_R subunits are encoded by three genes in vertebrates. The three large, closely related subunits assemble into homo- and hetero-tetrameric structures, which form large-conductance Ca^2+^-permeable channels within intracellular membranes, primarily those of the endoplasmic reticulum [10]. Opening of the central pore is initiated by binding of IP_3_ to all four IP_3_R subunits [15], which evokes conformational changes within the N-terminal domains of the IP_3_R [16]. These conformational changes are proposed to facilitate binding of Ca^2+^, which then triggers opening of the pore. Hence, the IP_3_R is itself a coincidence detector, responding only when provided with both cytosolic IP_3_ and Ca^2+^. High-resolution structures of the N-terminal region of an IP_3_R with and without IP_3_ bound [16], and cryo-electron microscopy reconstructions of the entire IP_3_R in a closed state [17] have begun to reveal the workings of the IP_3_R machinery. However, the mechanisms linking IP_3_ binding to channel gating are not yet fully resolved. While IP_3_ and Ca^2+^ are the essential regulators of IP_3_R gating, many additional signals modulate IP_3_R behaviour [18]. My focus on cAMP therefore provides only a rather restricted view of the capacity of IP_3_Rs to integrate information provided by different signalling pathways.

## 2 Regulation of IP_3_Rs by PKA

Cyclic AMP-dependent protein kinase (protein kinase A, PKA), exchange proteins activated by cAMP (EPACs), cyclic nucleotide-activated cation channels (CNGs), and some cyclic nucleotide phosphodiesterases (PDEs) are the major targets of cAMP in mammals. At least some of these targets regulate IP_3_-evoked Ca^2+^ signalling. PKA, for example, stimulates Ca^2+^ uptake into the sarcoplasmic reticulum, and EPACs through the small G protein rap2B stimulate PLCε [11]. However, only PKA has been convincingly shown to interact directly with IP_3_Rs. The three IP_3_R subtypes are closely related, but each has a distinctive distribution of PKA phosphorylation sites. The many effects of cAMP within Ca^2+^ signalling pathways were sources of some confusion in the pioneering studies of IP_3_R phosphorylation [19], but the consensus now is that PKA-mediated phosphorylation of IP_3_R1 and IP_3_R2 enhances their activity, while the functional significance of such phosphorylation for IP_3_R3 is less clear [14], [20].

Two residues (S^1589^ and S^1755^) within the central cytosolic domain of IP_3_R1 are phosphorylated by PKA, and their replacement by non-phosphorylatable alanine residues confirms that they are the only sites [21]. Phosphorylation of IP_3_R1 by PKA or introduction of phosphomimetic residues (S^1589^E/S^1755^E) do not themselves open the channel, but they increase the open probability (*P*_o_) of channels activated by IP_3_. The increased *P*_o_ results from shortening of the gaps between bursts of channel openings and an increase in the duration of the bursts, with no obvious effect on IP_3_ binding or the sensitivity to Ca^2+^ regulation [22]. Hence, phosphorylation of IP_3_R1 by PKA improves the coupling of IP_3_ and Ca^2+^ binding to channel gating by both stabilizing the bursting state of the IP_3_R and destabilizing a prolonged closed state. An alternative splice site (S2, residues 1693–1732), which encodes 40 residues and is removed from non-neuronal IP_3_R1, abuts the second phosphorylation site (S^1755^). For the neuronal S2^+^ form of IP_3_R1, S^1755^ entirely mediates the effects of PKA, while in the peripheral S2^−^ form both residues (S^1589^ and S^1755^) must be phosphorylated for PKA to enhance IP_3_-evoked Ca^2+^ release [23]. Effective phosphorylation and dephosphorylation of IP_3_R1 are facilitated by tethering of PKA to IP_3_R1 by AKAP9 (A-kinase-anchoring protein 9) [24] and of the protein phosphatase, PP1α, by IRBIT [25], AKAP9 or directly to the C-terminal tail of IP_3_R1 [26].

The consensus sequences for PKA and cGMP-dependent protein kinase (PKG) are similar, such that some residues (e.g. S^1755^ in IP_3_R1S2^+^) are phosphorylated by either kinase. Yet in native tissues PKG and PKA often exert opposing effects on IP_3_-evoked Ca^2+^ release. The difference may, at least in part, be due to expression of IRAG (IP_3_R-associated cGMP kinase substrate), which blocks phosphorylation of IP_3_R1 by PKA, and IRAG phosphorylated by PKG inhibits IP_3_R [27]. Hence, IRAG diverts PKG from the PKA-phosphorylation sites and imposes its own inhibition. PKA also modulates the interaction of IP_3_R1 with its endogenous antagonist, IRBIT (IP_3_R-binding protein released by IP_3_), apparently decreasing the affinity for IRBIT so that IP_3_ more effectively competes for occupancy of their shared binding site on the IP_3_R [28]. Hence in secretory epithelia, receptors that stimulate formation of cAMP and IP_3_ synergistically stimulate release of IRBIT from IP_3_Rs, and IRBIT then directly stimulates two of the ion transporters that sustain fluid transport [28].

Long before the discovery IP_3_Rs, synergistic stimulation of a Ca^2+^-sensitive K^+^ channel by α_1_-adrenoceptors (which stimulate PLC) and β-adrenoceptors (which stimulate formation of cAMP) in hepatocytes suggested that cAMP might enhance receptor-mediated Ca^2+^ release from intracellular stores [29]. Subsequent studies confirmed that PKA stimulates phosphorylation of hepatic IP_3_Rs [30] and potentiates IP_3_-evoked Ca^2+^ release [31], [32]. IP_3_R2, the major IP_3_R subtype in hepatocytes, is phosphorylated by PKA at a single residue (Ser^937^), although others suggest that IP_3_R2 is a rather poor substrate for PKA [20]. Ser^937^ is unique to IP_3_R2, but the functional consequences of the phosphorylation appear similar to those seen with IP_3_R1, namely enhanced bursts of IP_3_R gating [33]. Additional effects of PKA, including an increase in IP_3_ binding affinity [30] and recruitment of IP_3_Rs into functional Ca^2+^ stores [32], may also contribute to the effects of PKA on IP_3_R2 in intact cells.

The effects of PKA on IP_3_R3 have been least explored. In intact cells, IP_3_R3 is phosphorylated by PKA at three sites (S^916^, S^934^, S^1832^) that are unique to IP_3_R3, with S^934^ being the most extensively phosphorylated [34]. But, at least in cells expressing only IP_3_R3, PKA has no effect on IP_3_-evoked Ca^2+^ release triggered by cell-surface receptors [34]. Whether the phosphorylation affects other aspects of IP_3_R3 behaviour remain to be determined.

## 3 Direct regulation of IP_3_Rs by cAMP

**Fig. 1:**
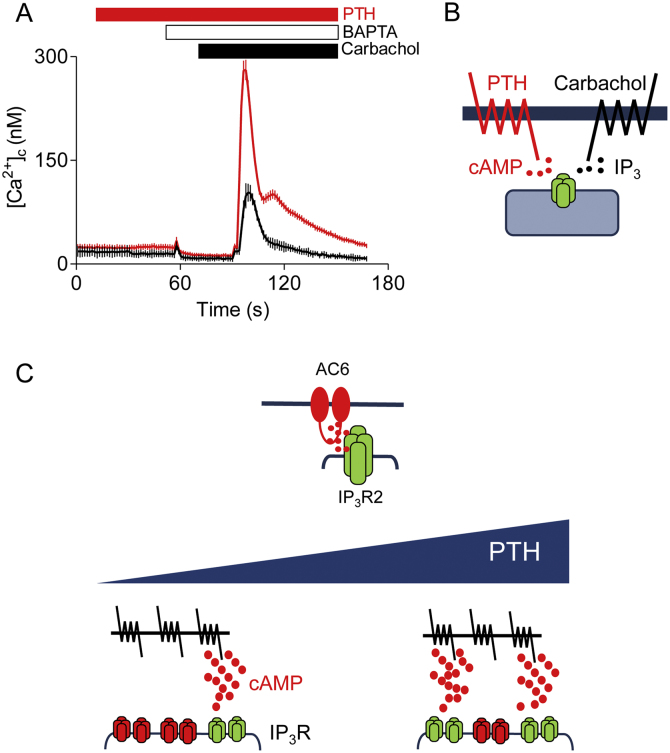
Regulation of IP_3_Rs by cAMP signalling junctions. (A) Populations of fluo4-loaded HEK-293 cells stably expressing type 1 receptors for PTH were stimulated with PTH (100 nM, red line) and carbachol (20 μM, both lines) as indicated after addition of BAPTA to chelate extracellular Ca^2+^. Results show that PTH does not alone evoke an increase in [Ca^2+^]_c_, but it potentiates the Ca^2+^ signal evoked by carbachol. Similar results are shown in Refs. [36], [39]. (B) In HEK-293 cells, PTH through heterologously expressed type 1 PTH receptors stimulates adenylyl cyclase and so formation of cAMP. Carbachol stimulates endogenous M_3_ muscarinic receptors, which activate PLC and thereby formation of IP_3_ and release of Ca^2+^ from the ER through IP_3_Rs. The potentiation of carbachol-evoked Ca^2+^ signals by PTH is entirely mediated by cAMP, which enhances IP_3_-evoked Ca^2+^ release from IP_3_Rs. (C) IP_3_R2 and AC6 are selectively associated at cAMP signalling junctions. Within these junctions, cAMP is delivered from AC to IP_3_Rs at concentrations far greater than needed to maximally sensitize the associated IP_3_Rs. This allows each junction to function as a digital ‘on-off’ switch, it ensures that cAMP-mediated signalling operates with a considerable safety margin, and it allows rapid activation of the associated IP_3_Rs (as cAMP is locally delivered at high concentration) and rapid de-activation (as diffusion of cAMP form the junction reduces its concentration to below that needed for sensitizing IP_3_Rs). Since each signalling junction operates as an ‘on-off’ switch, the concentration-dependent effects of PTH are proposed to be due to recruitment of junctions, rather than to graded activity within individual junctions.

In HEK-293 cells stably expressing human type 1 receptors for parathyroid hormone (PTH), PTH stimulates formation of cAMP, but it does not alone evoke an increase in cytosolic Ca^2+^ concentration ([Ca^2+^]_c_). However, PTH potentiates the increase in [Ca^2+^]_c_ evoked by receptors that stimulate PLC, the endogenous muscarinic M_3_ receptors of HEK-293 cells, for example, which can be activated by carbachol (Fig. 1A). This effect of PTH is mimicked by stimulation of endogenous prostanoid receptors or β-adrenoceptors, by direct activation of adenylyl cyclase with forskolin or by addition of a membrane-permeant analog of cAMP, 8-Br-cAMP. The non-additive effects of maximally effective concentrations of PTH and 8-Br-cAMP confirm that the effect of PTH on carbachol-evoked Ca^2+^ signals is entirely mediated by cAMP (Fig. 1B) [35], [36]. Responses to other PLC-coupled receptors are also potentiated by PTH, and the enhanced responses are not associated with increased production of IP_3_ [35], [37]. Furthermore, cAMP also potentiates the Ca^2+^ signals evoked by a membrane-permeant form of IP_3_ (IP_3_-BM) [38]. These results, demonstrating that cAMP acts downstream of IP_3_, are important because cAMP can, through EPACs, stimulate PLCε [11]. However, the effects of PTH are neither mimicked by EPAC-selective analogs of cAMP [36] nor blocked by an EPAC antagonist [39]. The enhanced IP_3_ −evoked increases in [Ca^2+^]_c_ are not due inhibition of Ca^2+^ extrusion from the cytosol by cAMP [38]. Furthermore, cAMP potentiates IP_3_-evoked Ca^2+^ release in permeabilized cells [40], and it enhances IP_3_-gated channel activity in nuclear patch-clamp recordings of IP_3_R [40]. These results, where cAMP potentiates the activation of IP_3_R by IP_3_, seem consistent with the many reports suggesting that phosphorylation of IP_3_R1 and IP_3_R2 by PKA enhances responses to IP_3_ (see preceding section). However, several lines of evidence demonstrate that this is not a sufficient explanation:

1. When PTH-evoked protein phosphorylation is blocked by inhibition of either PKA activity (using H89) or the association of PKA with A-kinase-anchoring proteins (AKAPs, using a membrane-permeant form of an uncoupling protein, ht-89), there is no effect on the ability of any concentration of PTH to potentiate the Ca^2+^ signals evoked by carbachol [36], [38], [39]. Others have also suggested that potentiation of carbachol-evoked Ca^2+^ signals by β_2_-adrenoceptors is insensitive to inhibition of PKA in HEK-293 cells [41]. Similar results were reported for rat osteoblasts, where potentiation of ATP-evoked Ca^2+^ signals by PTH was unaffected by inhibition of PKA [42].
2. In permeabilized HEK-293 cells, the catalytic subunit of PKA causes minimal phosphorylation of IP_3_R and a barely detectable increase in the sensitivity of the Ca^2+^ stores to IP_3_, while cAMP and 8-Br-cAMP cause substantial increases in IP_3_ sensitivity [36].
3. The concentrations of 8-Br-cAMP (in intact cells) and of cAMP (in permeabilized cells) needed to sensitize IP_3_Rs to IP_3_ are much higher than those required to activate PKA [36].
4. In DT40 cells expressing single IP_3_R subtypes, high concentrations of cAMP potentiate IP_3_-evoked Ca^2+^ release through IP_3_R1, IP_3_R2 and IP_3_R3 [40]. This does not align with the consensus that PKA increases the sensitivity of only IP_3_R1 and IP_3_R2 [14].
5. In permeabilized DT40 cells expressing IP_3_R2, cAMP potentiates the Ca^2+^ release evoked by IP_3_, and the effect of cAMP is unaffected by addition of either H89 (to inhibit PKA) or the catalytic subunit of PKA.
6. In nuclear patch-clamp recordings from IP_3_R2 expressed in DT40 cells and stimulated with IP_3_, cAMP increases channel activity in the absence of ATP [40], confirming that protein phosphorylation is not required.

These observations suggest that cAMP can regulate IP_3_R activity via both PKA and by mechanisms that do not require activation of either PKA or EPACs. The observations are intriguing because they suggest an effect of cAMP that is not mediated by any of its conventional targets. Our results indicate that while cAMP alone cannot activate IP_3_Rs and nor does cAMP affect IP_3_ binding to IP_3_Rs [40], it does enhance the effectiveness with which the essential co-agonists, IP_3_ and Ca^2+^, stimulate channel opening. The mechanisms underlying these non-canonical actions of cAMP are not yet resolved. In light of evidence that the effects of cAMP are preserved in isolated nuclei and permeabilized cells [36], [40], it seems likely that binding of cAMP to a low-affinity site within either the IP_3_R itself or a tightly associated protein mediates this allosteric regulation of IP_3_Rs by cAMP.

## 4 Signalling to IP_3_Rs at cAMP junctions

Despite compelling evidence that cAMP entirely mediates the potentiating effects of PTH on IP_3_-evoked Ca^2+^ signals [36], there are some puzzling features of the signalling pathway that initially led us to a different conclusion. Firstly, the direct effects of cAMP on IP_3_Rs require much higher concentrations of cAMP than are needed for activation of PKA or EPACs, and probably much higher than the average concentrations achieved in stimulated cells [36]. Secondly, although many different stimuli evoke cAMP formation and potentiation of carbachol-evoked Ca^2+^ signals, the relationship between their effects on cAMP and Ca^2+^ are entirely different for different stimuli. For example, for concentrations of PTH and isoproterenol (which activates β-adrenoceptors) that cause similar submaximal potentiation of Ca^2+^ signals, PTH evokes a more than 10-fold greater increase in intracellular cAMP concentration than does isopreterenol [36]. This immediately suggests that the cAMP that regulates IP_3_R activity cannot be uniformly distributed in the cytosol. Thirdly, and more troublesome, are the many manipulations of cAMP formation that fail to affect the carbachol-evoked Ca^2+^ signals. Hence substantial inhibition of cAMP formation by either low-affinity inhibitors of adenylyl cyclase (AC, ∼90% inhibition in Ref. [38] and ∼70% in Ref. [36]) or siRNA-mediated knockdown of AC3 (the major subtype in HEK-293 cells), or an enhancement of cAMP accumulation after inhibition of cyclic nucleotide phosphodiesterase [36], [38], [39] had no effect on the ability of any concentration of PTH to potentiate carbachol-evoked Ca^2+^ signals. This initially led us to conclude that the effects of PTH were not mediated by cAMP [38], but we had then to revise that conclusion in light of evidence that cAMP *does* mediate the effects of PTH.

It is easy to envisage how the effects of a maximal concentration of PTH might be unaffected by even very substantial inhibition of AC if there are ‘spare receptors’, such that maximal activation of the receptors can provide more cAMP than needed to cause maximal activation of IP_3_Rs. However, that argument cannot be employed to explain the lack of effect of AC inhibitors on the Ca^2+^ responses evoked by *submaximal* concentrations of PTH. We therefore proposed that the ‘spare’ signalling capacity might exist within subcellular compartments or ‘signalling junctions’. We suggest that cAMP is delivered to IP_3_Rs locally at concentrations substantially greater than required to cause maximal sensitization of the associated IP_3_Rs (Fig. 1C). The concentration-dependent effects of PTH, we suggest, come from recruitment of these hyperactive signalling junctions, rather than from graded activity within individual junctions. Each signalling junction is, in effect, an ‘on-off’ switch with a considerable safety margin because once activated it delivers more cAMP than needed to fully sensitize the associated IP_3_Rs. Our scheme neatly accounts for both the inconsistent relationship between cAMP and response for different stimuli (because different stimuli operate with different safety margins) and it provides a mechanism that would allow IP_3_Rs to be exposed to high concentrations of cAMP. It also accommodates the results showing that even manipulations of cAMP concentration fail to effect the Ca^2+^ signals evoked by PTH (because the large safety margin protects the signalling pathway from even substantial perturbations of cAMP).

The involvement of signalling junctions is supported by additional evidence [36]. Notably, there is a selective association between AC6 (which accounts for only 5% of AC in HEK-293 cells) and IP_3_R2 in HEK-293 cells, consistent with targeted delivery of cAMP from AC to IP_3_R. Loss of IP_3_R2 (using siRNA) selectively attenuates the potentiation of carbachol-evoked Ca^2+^ signals by PTH. Global inhibition of AC activity by low-affinity inhibitors reduces PTH-evoked cAMP formation without affecting Ca^2+^ signals, whereas the converse occurs when expression of AC6 is reduced. Loss of AC6 has no perceptible effect on cAMP levels, but it attenuates the potentiation of carbachol-evoked Ca^2+^ signals by PTH. The rationale, we suggest, is that all signalling junctions feel the effect of the low-affinity inhibitors, which thereby reduces the cAMP delivered within junctions but not sufficiently to obliterate the safety margin, whereas removing AC6 from individual junctions (each perhaps containing only a single AC) will incapacitate that junction. Finally, in cells with diminished expression of αs, which couples receptors to AC, the safety margin is reduced such that further inhibition of AC (using the low-affinity AC inhibitors) does reduce the ability of PTH to potentiate carbachol-evoked Ca^2+^ signals [36].

In conclusion, there are at least two routes through which cAMP can directly modulate IP_3_R gating. Phosphorylation of IP_3_R1 and IP_3_R2 by PKA increases the effectiveness with which IP_3_ and Ca^2+^ evoke bursts of channel openings. In addition, binding of cAMP to a low-affinity site that seems to be closely associated with the IP_3_R can also increase the apparent efficacy of IP_3_ and Ca^2+^ in gating each IP_3_R subtype. The need for high concentrations of cAMP for this direct action demands local delivery of cAMP to IP_3_Rs, and that has so far been shown to occur for only IP_3_R2 [36]. The low-affinity of IP_3_Rs for cAMP effectively insulates them from global changes in cytosolic cAMP concentration and allows them to respond only to cAMP delivered to them within signalling junctions (Fig. 1C). Because each active junction delivers cAMP at a super-saturating concentration to associated IP_3_Rs, the junction behaves as a robust digital switch that can rapidly respond to changes in extracellular stimulus intensity. The cAMP is delivered rapidly and at a high concentration driving rapid association of cAMP with IP_3_Rs, and as soon as the AC stimulus is removed the focal concentration of cAMP dissipates by diffusion, rapidly terminating the effects of cAMP on IP_3_Rs.
